# Caveolin-1 and CDC42 mediated endocytosis of silica-coated iron oxide nanoparticles in HeLa cells

**DOI:** 10.3762/bjnano.6.16

**Published:** 2015-01-14

**Authors:** Nils Bohmer, Andreas Jordan

**Affiliations:** 1 Project Biomedical Nanotechnologies, Charité University Medicine, 13353 Berlin, Germany; 2 MagForce Nanotechnologies AG, 12489 Berlin, Germany

**Keywords:** Caveolin-1, CDC42, endocytosis inhibition, iron oxide nanoparticles, nanoparticle uptake

## Abstract

Nanomedicine is a rapidly growing field in nanotechnology, which has great potential in the development of new therapies for numerous diseases. For example iron oxide nanoparticles are in clinical use already in the thermotherapy of brain cancer. Although it has been shown, that tumor cells take up these particles in vitro, little is known about the internalization routes. Understanding of the underlying uptake mechanisms would be very useful for faster and precise development of nanoparticles for clinical applications. This study aims at the identification of key proteins, which are crucial for the active uptake of iron oxide nanoparticles by HeLa cells (human cervical cancer) as a model cell line. Cells were transfected with specific siRNAs against Caveolin-1, Dynamin 2, Flotillin-1, Clathrin, PIP5Kα and CDC42. Knockdown of Caveolin-1 reduces endocytosis of superparamagnetic iron oxide nanoparticles (SPIONs) and silica-coated iron oxide nanoparticles (SCIONs) between 23 and 41%, depending on the surface characteristics of the nanoparticles and the experimental design. Knockdown of CDC42 showed a 46% decrease of the internalization of PEGylated SPIONs within 24 h incubation time. Knockdown of Dynamin 2, Flotillin-1, Clathrin and PIP5Kα caused no or only minor effects. Hence endocytosis in HeLa cells of iron oxide nanoparticles, used in this study, is mainly mediated by Caveolin-1 and CDC42. It is shown here for the first time, which proteins of the endocytotic pathway mediate the endocytosis of silica-coated iron oxide nanoparticles in HeLa cells in vitro. In future studies more experiments should be carried out with different cell lines and other well-defined nanoparticle species to elucidate possible general principles.

## Introduction

Nanotechnology is expected to be a very powerful technique for the treatment of various diseases in the 21st century. Today nanomedicine has spread in many different subareas, which are working highly interdisciplinary on the development of new therapy concepts [1].

One of the most important fields is the early detection and treatment of cancer. Therefore many strategies and specific nanoparticle constructs have been explored in recent years [2–4], although only few of them have already made their way into practice [5]. Iron oxide nanoparticles are of special interest because of their magnetic properties, which make them suitable for clinical applications. Nowadays they are already in use in magnetic resonance imaging [6–8] and the thermotherapy of tumors [9–11]. Also for the investigation of applications in theragnostic and drug delivery iron oxide nanoparticles are promising tools for the future [12–18].

Despite the commercial use of iron oxide nanoparticles and their diversified development for future applications in nanomedicine, little is known about the way they are internalized by tumor cells or cells of other origins. By the use of microscopic techniques previous studies showed, that iron oxide nanoparticles often appear in vesicular structures within the cytosol of cells in vitro [19–24], which indicates an active, energy dependent uptake via endocytosis. In a post mortem study of glioma patients, who had received thermotherapy with aminosilane coated iron oxide nanoparticles in a phase-II study, nanoparticles were mostly found in macrophages than in the cancer cells themselves [25]. In the respective study it was not crucial for successful treatment that the nanoparticles were specifically taken up by the tumor cells, because they were injected directly into the tumor and had no further payload attached to the surface. But for drug delivery applications and intravenous injections it would be very useful to understand, how cancer cells internalize iron oxide nanoparticles and which pathways are involved. Insights in the principles of nanoparticle endocytosis would be very helpful to develop nanoparticle species, which are taken up specifically by target cells and exploit their maximum potential.

**Figure 1:**
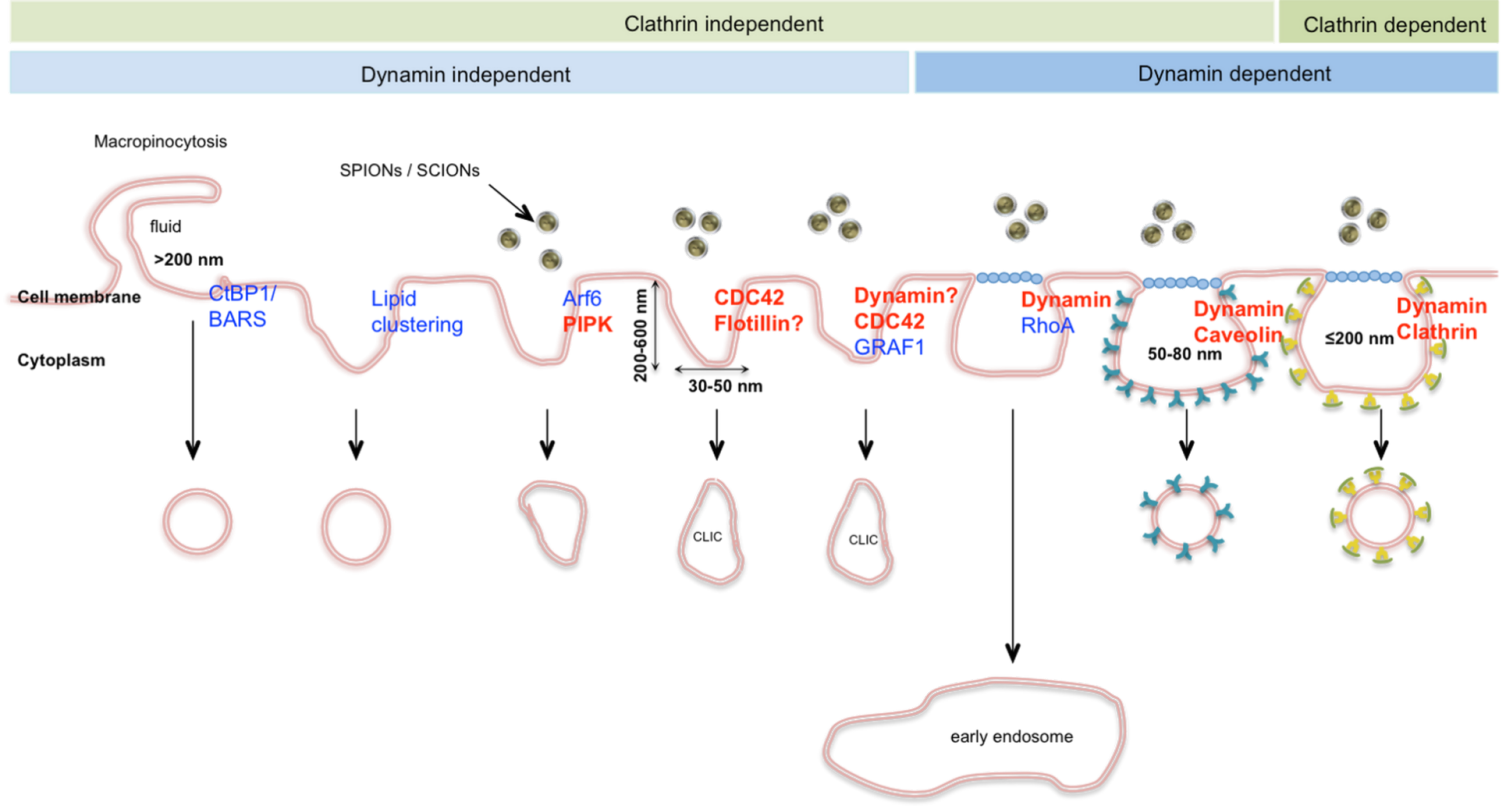
Overview of well-known endocytotic pathways and the involved key proteins, target proteins inhibited in this study are marked in red (adapted from Wieffer et al. [27], sizes of membrane ruffling from Canton et al. [28]).

In this study differently modified silica coated superparamagnetic iron oxide nanoparticles (SPIONs) and silica coated iron oxide nanoparticles (SCIONs), which were all comparable in their primary size and surface charge, were tested in HeLa cells as a model cell line. To elucidate, which molecular pathways are involved in their endocytosis, well-known endocytotic mechanisms [26–28] were inhibited by specific knockdown of key proteins via siRNA (Figure 1).

## Experimental

### Superparamagnetic iron oxide nanoparticles (SPIONs)

**Figure 2:**
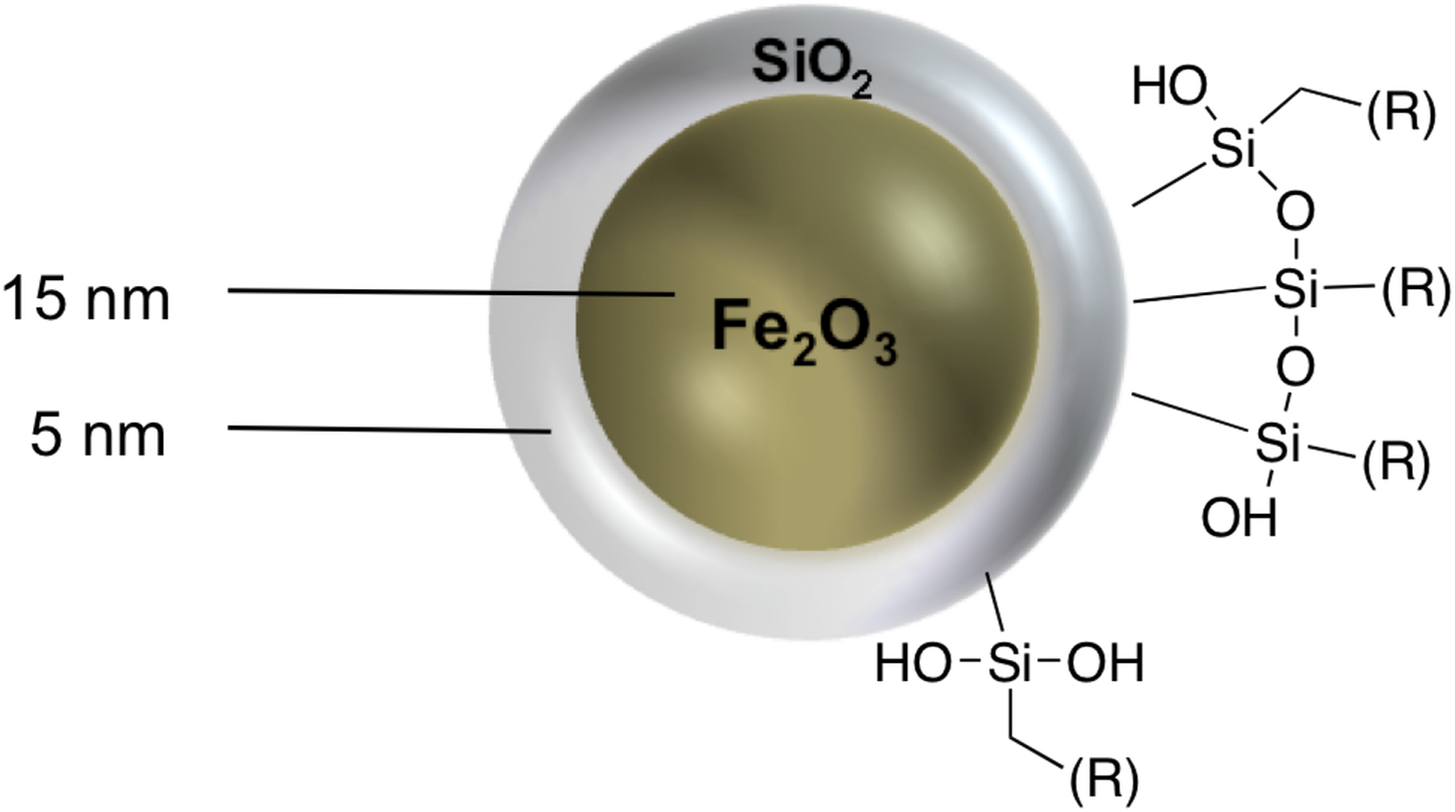
Schematic overview of SPION structure.

SPIONs were provided and characterized by MagForce AG. SPIONs with an iron oxide core of 15 nm and a silica shell of 5 nm were modified by coupling the respective functional groups as an ethoxy- or rather methoxysilane to the free hydroxy groups of the surface (Figure 2). These modifications resulted in different physicochemical properties referring to SPIONs surface charge and their size distribution under physiological conditions (Table 1). The primary particle size was determined by transmission electron microscopy (EM906, Zeiss). The zeta potential and the average hydrodynamic diameter in physiological environment were measured by dynamic light scattering (Zetasizer, Malvern Instruments Ltd). Due to the synthesis route, often more than one iron core was enclosed during growth of the silica shells. This caused aggregation and therefore the nanoparticle suspensions were polydisperse.

**Table 1:** Surface modification of SPIONs and their physical properties at room temperature in aqueous dispersion (pH 7, DLS = dynamic light scattering).

Surface modification	Linker structure	Zeta potential	Average diameter (DLS)

None (pure silica)	—	−59 mV	136 nm
Carboxylic acid	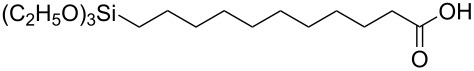	−47 mV	157 nm
Polyethylene glycol	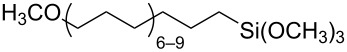	−14 mV	133 nm

The tested modifications were carboxylic acid- and PEG- silanes (Cat. No. SIC2264.0 and SIM6492.7, ABCR GmbH & Co. KG, Karlsruhe, Germany). SPIONs had been sterilized and pyrogen tested by MagForce AG. They were stored at 4 °C in aqueous suspension with an iron concentration of 34 mg/mL.

### Fluorescent silica coated iron oxide nanoparticles (SCIONs)

SCIONs were provided and characterized by the National Institute of Health (Maryland, USA). They were monodisperse at pH 7 and had a hydrodynamic diameter of 17 nm with a surface charge of 50 ± 5 mV. For detection in confocal fluorescence microscopy, the fluorescent dye Alexa Fluor^®^ 555 was embedded into their shells. Further details of synthesis and characterization have been described elsewhere [29–30].

#### Cell culture

HeLa cells (human cervix carcinoma) were provided by the group of Professor Haucke (Freie Universität, Berlin, Germany). They were grown in Dulbecco`s Modified Eagle Medium (DMEM, Invitrogen, Cat. No. 31885023), supplemented with 10% FBS, and cultivated in an incubator at 37 °C and 5% CO_2_.

### Transfection procedure and efficiency

#### Lipofectamine™ 2000 transfection reagent

Cells were transfected according to the standard protocol of Life Technologies. To achieve the optimal transfection efficiency, two transfection rounds on day 1 and 3 after cell plating were performed. In preliminary experiments the knockdown technique was optimized to cause no cell death by applying different ranges of transfection reagent with different amounts of siRNA. The siRNAs were purchased from Eurofins MWG Operon (Ebersberg, Germany). The sequences are displayed in Table 2. With the exception of Flotillin-1 all sequences where created and established by the group of Professor Haucke (Freie Universität, Berlin, Germany).

**Table 2:** Sequences of siRNAs used for transfection with Lipofectamine™ 2000 transfection reagent.

Oligo name (siRNA)	Oligo details (sense strand)

Flotillin-1^a^	5’-CACACUGACCCUCAAUGUC-3’
Caveolin-1	5’-CCUGAUUGAGAUUCAGUGC-3’
Clathrin heavy chain	5’-AUCCAAUUCGAAGACCAAUTT-3’
Dynamin 2	5’-GCAACUGACCAACCACAUC-3’
Nonsense control	5’-GUAACUGUCGGCUCGUGGUTT-3’

^a^Sequence from Glebov et al. [31].

#### Dharmacon SMARTpool^®^ technology

Cells were transfected according to the protocol “Thermo Scientific DharmaFECT Transfection Reagents - siRNA Transfection Protocol“ and DharmaFECT 1 siRNA Transfection Reagent (Thermo Fisher Scientific, Cat. No. T-2001-01) was used. Information about the siRNA-mix (SMARTpool^®^) used is shown in Table 3.

**Table 3:** siRNA-pools used to transfect HeLa cells (Dharmacon SMARTpool^®^).

SMARTpool^®^	Catalog number

ON-TARGETplus SMARTpool-Human PIP5K2α	L-006778-00-0005
ON-TARGETplus SMARTpool-Human CDC42	L-005057-00-0005

#### Knockdown efficiency

To demonstrate effective knockdown of target proteins, transfected cells were collected in every single experiment. The expression level of target proteins was determined in comparison to non-transfected control cells by sodium dodecyl sulfate polyacrylamide gel electrophoresis (SDS-PAGE) of cell lysates followed by Western Blot and detection of proteins through specific antibodys. ß-Actin served as a housekeeping protein to ensure comparable amounts of proteins in every sample. Primary antibodies used in this study are shown in Table 4.

**Table 4:** Primary antibodies with source and dilution used for detection of target proteins.

Antibody	Source	Catalog number	Dilution

Caveolin-1 (N20)	Santa Cruz Biotechnology	sc-894	1:400
Purified mouse Flotillin-1	BD Transduction	610820	1:100
Cdc42 polyclonal	Thermo Scientific	PA5-17544	1:1000
Monoclonal anti-PIP5K2A	Sigma-Aldrich	WH0005305M1-100UG	1:350
Monoclonal anti-ß-actin	Sigma-Aldrich	A5441-2ML	1:5000
Anti-Dynamin II polyclonal	Thermo Scientific	PA1-661	1:1000
Antibody Clathrin	Haucke group (FU Berlin, Germany)	unknown	1:500

#### Nanoparticle exposure

Cells were grown to 70–80% confluence. Before every single experiment, nanoparticles were prewarmed to 37 °C and treated with ultrasound for 10 min to avoid sedimentation and aggregation. SPIONs were diluted with cell culture media to a concentration of 50 µg Fe/mL while SCIONs were diluted to 5 µg/mL. The concentration of the SCIONs was chosen after preliminary experiments. It could be shown that 5 µg Fe/mL provides the lowest background fluorescence combined with a good intracellular signal. In both setups the cell culture media contains SCIONs in excess to the internalization rate of the cells. Afterward cells were exposed to nanoparticles for 24 h (SPIONs) or 4 h (SCIONs). To ensure natural behavior of nanoparticles in cell culture media, the plates were left without any movement or shaking during the exposition. During the incubation time of cells with nanoparticles no or only minor differences of the cell numbers were observed, which confirmed no severe impact of the treatment on cell viability within the observation period.

#### Quantitative iron analysis

For quantitative determination of iron, which was taken up by cells and not attached to the plastic surface or to the outer cell membrane, cells were washed two times with prewarmed cell culture medium with all additives. Preliminary tests had shown, that full medium at 37 °C removed extracellular adhered nanoparticles very effective compared to PBS and medium without additives. To remove the contaminative iron, cells were rinsed thoroughly with full medium without detaching cells from the culture surface. Afterward cells were detached with trypsin/EDTA, counted and cell pellets were resuspended in concentrated hydrochloric acid. Treatment with hydrochloric acid and ultrasound for 10 min destroyed the cells and dissociated the iron cores of SPIONs. The iron content of the samples was then determined by a photometric assay (Spectroquant^®^, Merck) and by ICP–MS (iCAP 6000, Thermo Scientific). Experiments were repeated three times.

#### Fluorescence microscopy

Spinning disk confocal microscopy was used to detect SCIONs inside cells. The applied system was the Zeiss Axiovert 200M-based spinning disc confocal microscope (PerkinElmer Life Sciences Inc., MA, USA). Microscopy and quantitative analyses were performed with the software Volocity (Improvision Inc.). For quantitative determination of SCIONs per cell at least 140 single cells were analyzed in single layers in each experiment. To calculate an average fluorescence intensity of SCIONs for a whole cell population, the overall fluorescence of the dye Alexa Fluor^®^ 555 in every single cell was determined. Therefore the sensitivity of the nanoparticle channel was adjusted to the distinct vesicles containing SCIONs. To exclude background fluorescence of extracellular adherent SCIONs, single cells were marked by the help of fluorescently labeled transferrin (Transferrin From Human Serum, Alexa Fluor^®^ 488 Conjugate, Invitrogen, Cat.No. T-13342). After that the mean intensity of the SCION fluorescence per cell was determined. This procedure was the same for every sample. Experiments were repeated four times.

#### Statistics

To proof significance of detected differences between two populations, unpaired two-tailed t-tests (confidence interval γ = 95%, *p* value <0.05) were performed by the help of the software GraphPad Prism 5 (GraphPad Software Inc.).

## Results

### Transfection efficiency

**Figure 3:**
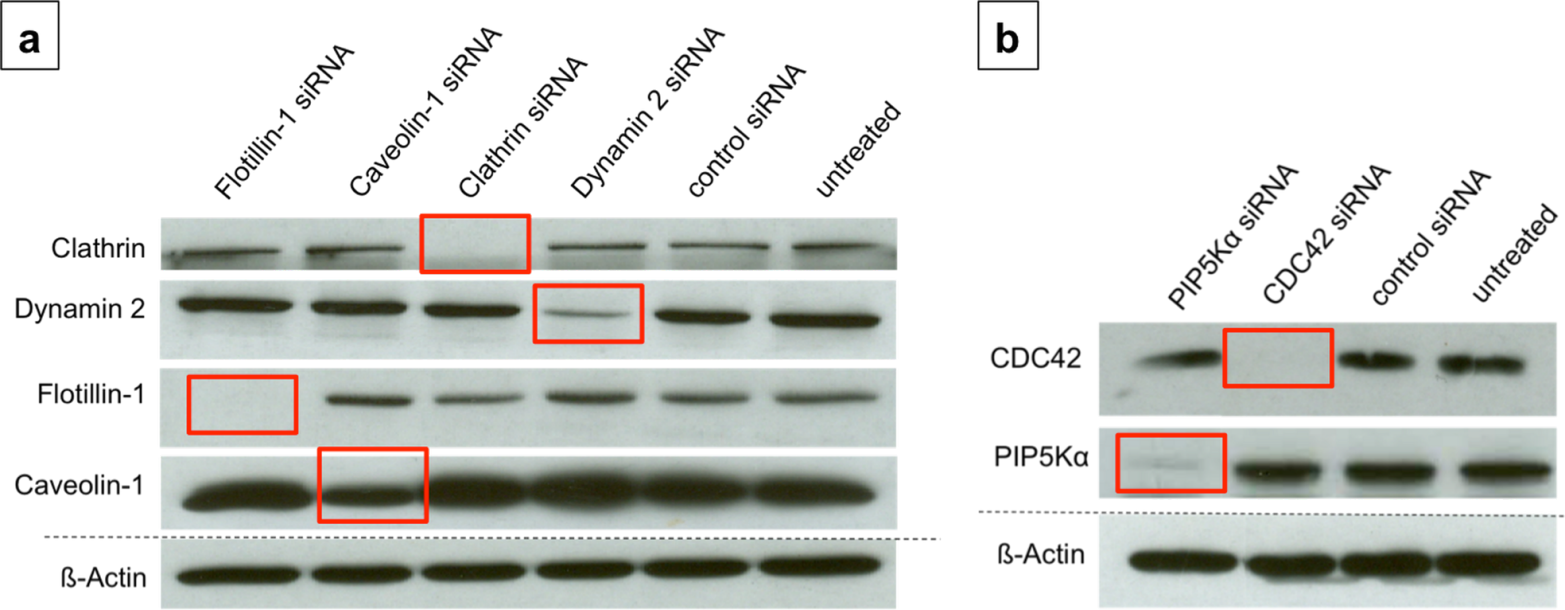
Representative X-ray films with knockdown efficiency of target proteins (red labels), ß-Actin serves as the protein loading control for comparable protein contents in the horizontal lines, control siRNA = siRNA with nonsense sequence, untreated = control cells without siRNA treatment; (a) Knockdown efficiency of Flotillin-1, Caveolin-1, Clathrin and Dynamin-2; (b) Knockdown efficiency of CDC42 and PIP5Kα.

Knockdown of target proteins was confirmed by determination of the expression level of the respective proteins in transfected and non-transfected cells in every single experiment. Semi-quantitative determination of proteins in cell lysates showed efficient knockdown for Clathrin, Dynamin 2, Flotillin-1, PIP5Kα and CDC42 (Figure 3). In case of Caveolin-1 around 50–60% knockdown efficiency has been achieved (Figure 3a). This was sufficient to detect an effect on the endocytosis of nanoparticles. ß-Actin was used as a housekeeping protein to ensure comparable amounts of proteins in every sample.

### Knockdown of Caveolin-1, Flotillin-1 and Clathrin

Endocytosis through Caveolin-1, Flotillin-1 and Clathrin was inhibited by knockdown of the respective protein by specific siRNAs via the Lipofectamine™ technology. HeLa cells were incubated for 24 h with SPIONs (50 µg Fe/mL) which were either caboxylated, PEGylated or which had no further modifications on their silica shell. Uptake of nanoparticles was measured by dissolution of cell pellets and nanoparticles in concentrated hydrochloric acid, followed by quantitative determination of the iron content by a photometric assay and ICP.

#### Bare SPIONs with silica shell

**Figure 4:**
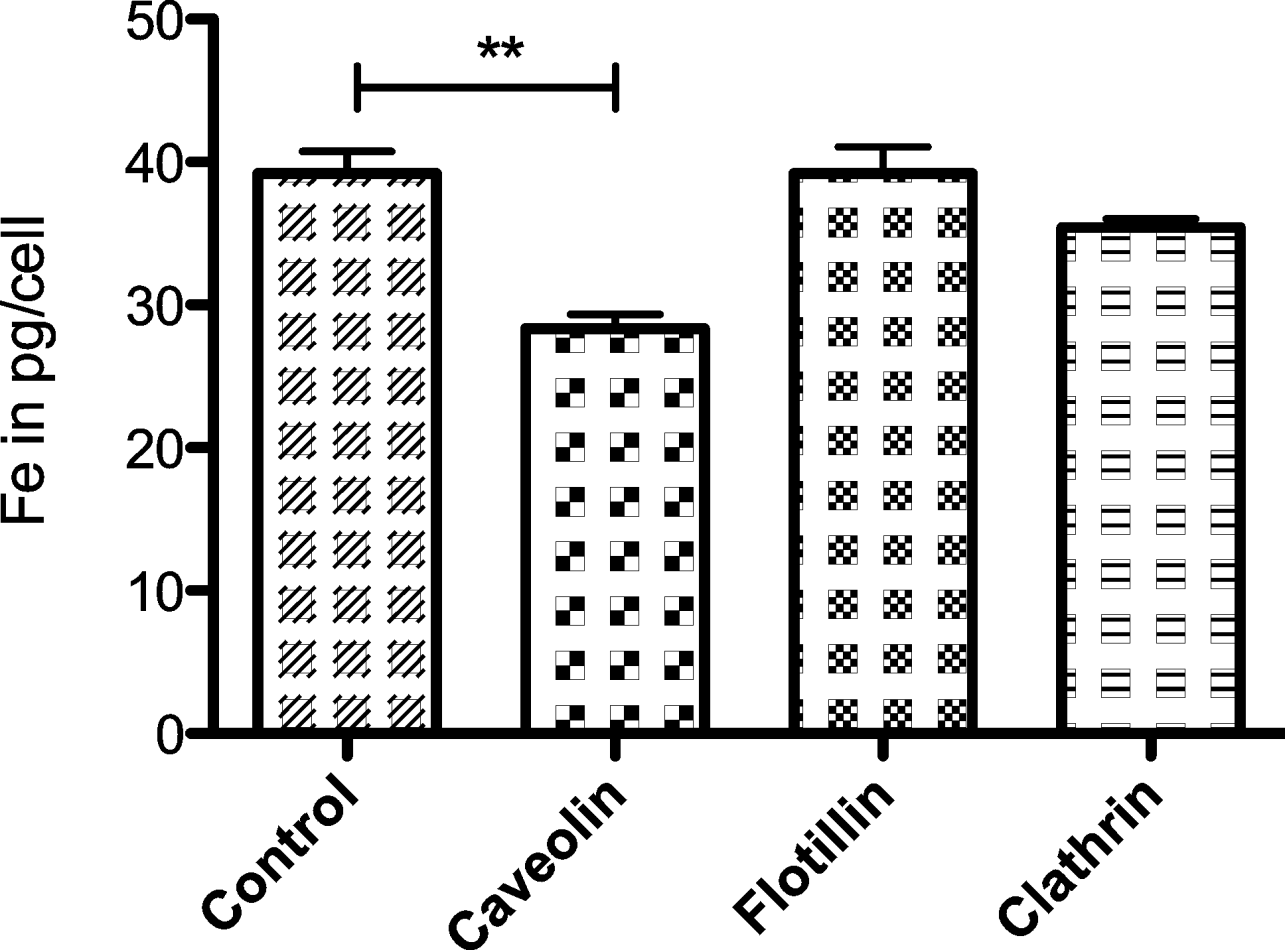
Iron content of control and transfected HeLa cells in pg/cell after 24 h incubation with unmodified SPIONs; target proteins: Caveolin-1, Flotillin-1, Clathrin (iron concentration 50 µg/mL, error bars: SEM, *n* = 3).

Non-transfected control cells internalized 39.2 ± 1.5 pg Fe/cell in 24 h, while cells with a knockdown of Caveolin-1 contained only 28.4 ± 1.0 pg Fe/cell. Hence, knockdown of Caveolin-1 decreased endocytosis of nanoparticles by 27% (Figure 4). This effect is statistical significant (γ = 95%, *p* = 0.0041). Knockdown of Flotillin-1 (39.2 ± 1.8 pg Fe/cell) and Clathrin (35.4 ± 0.6 pg Fe/cell) showed no significant difference in the iron content compared to control cells (Figure 4).

#### Carboxylated SPIONs

**Figure 5:**
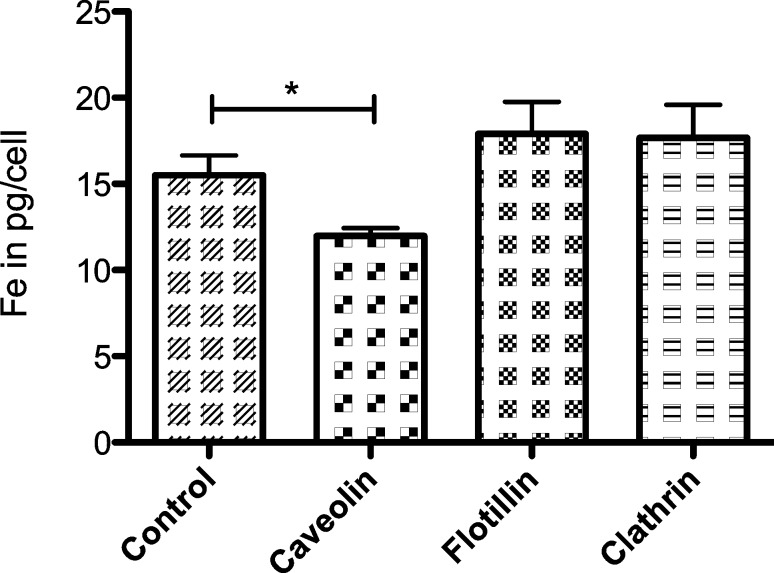
Iron content of control and transfected HeLa cells in pg/cell after 24 h incubation with carboxylated SPIONs; target proteins: Caveolin-1, Flotillin-1, Clathrin (iron concentration 50 µg/mL, error bars: SEM, *n* = 3).

HeLa cells with a knockdown of Caveolin-1 contained 23% less carboxylated SPIONs compared to non-transfected control cells (Figure 5). The iron levels per cell amounted to 12.0 ± 0.5 pg for Caveolin-1 depleted cells and 15.5 ± 1.2 pg for control cells. This effect is statistically significant (γ = 95%, *p* = 0.0466). Knockdown of Flotillin-1 (17.9 ± 1.9 pg Fe/cell) and Clathrin (17.7 ± 1.9 pg Fe/cell) resulted in slightly more nanoparticles inside the cells. However, this effect is statistically not significant (Figure 5).

#### PEGylated SPIONs

**Figure 6:**
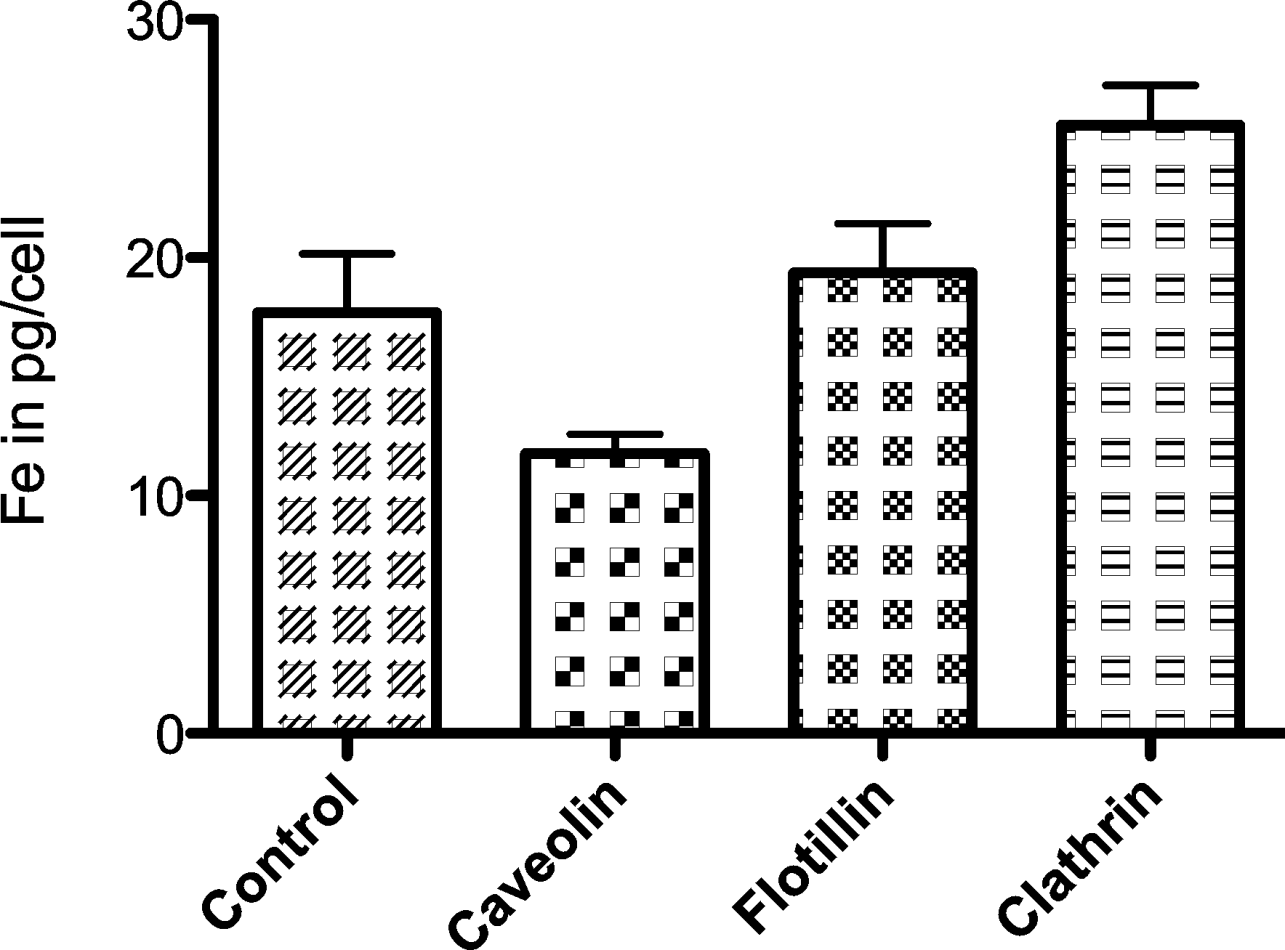
Iron content of control and transfected HeLa cells in pg/cell after 24 h incubation with PEGylated SPIONs; target proteins: Caveolin-1, Flotillin-1, Clathrin (iron concentration 50 µg/mL, error bars: SEM, *n* = 3).

Compared to control cells (17.7 ± 2.5 pg Fe/cell), HeLa cells contained 33% less iron (11.8 ± 0.8 pg Fe/cell) if the expression level of Caveolin-1 was reduced (Figure 6). Knockdown of Flotillin induced no detectable difference (19.4 ± 2.1 pg Fe/cell), while knockdown of Clathrin produced an elevated iron content per cell of 25.6 ± 1.7 pg and therefore an increase of 45% compared to control cells (Figure 6). All these effects are not statistically significant.

#### SCIONs in confocal fluorescence microscopy

**Figure 7:**
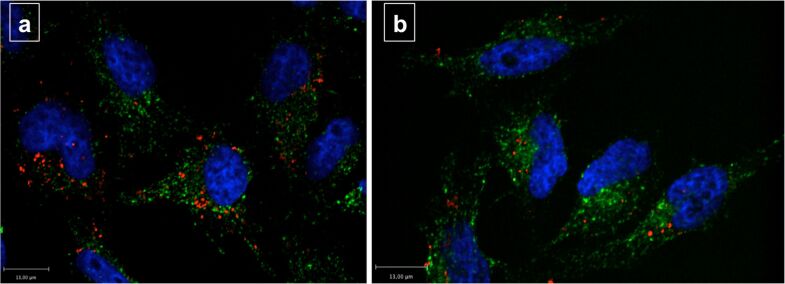
Fluorescence image of Hela cells which were incubated with SCIONs (iron concentration 5 µg/mL, incubation time 4 h), blue = DAPI (nuclei), green = Transferrin Alexa Fluor^®^ 488 conjugate (cytosol), red = Alexa Fluor^®^ 555 (SCIONs); (a) Control cells without siRNA treatment; (b) Cells with knockdown of Caveolin-1.

SCIONs were comparable to SPIONs in their primary size and surface charge. Because of the fluorescent dye Alexa Fluor^®^ 555, which was embedded into their cells, these nanoparticles were intracellular detectable with confocal fluorescence microscopy. To avoid heavy background fluorescence of extracellular adherent SCIONs, the iron concentration used was lowered to 5 µg/mL and the incubation time was shortened to 4 h. After incubation with nanoparticles, cells were imaged (at least 140 single cells per single experiment) and background fluorescence was eliminated (Figure 7). Finally, the average sum-fluorescence-intensity of SCIONs per cell was calculated.

**Figure 8:**
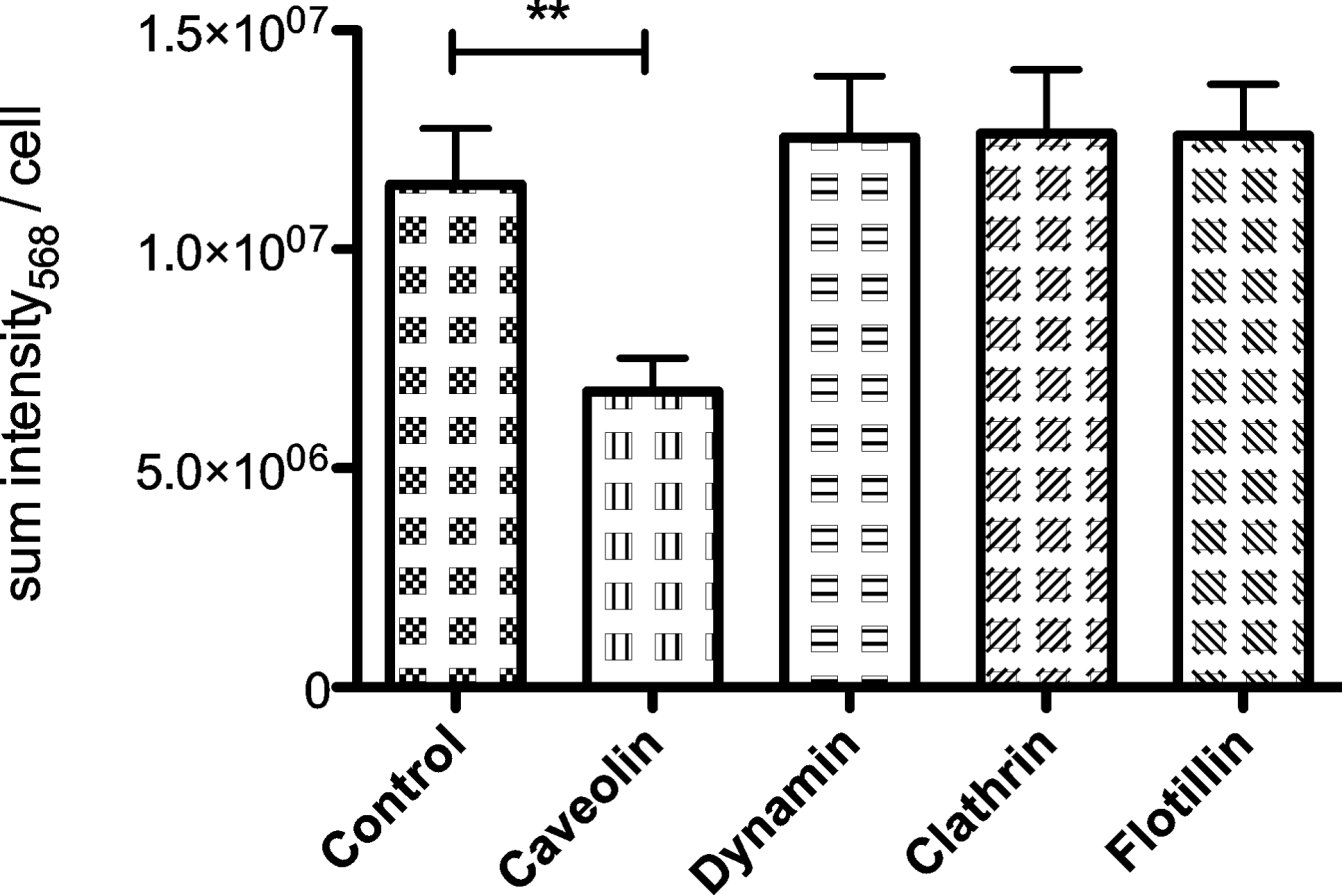
Sum-fluorescence-intensity (wavelength 568) per cell of SCIONs labeled with Alexa Fluor^®^ 555 in transfected HeLa cells after 4 h incubation time; target proteins: Caveolin-1, Dynamin 2, Flotillin-1, Clathrin (iron concentration 5 µg/mL, *n* = 4).

6.8 × 10^6^ ± 0.8 × 10^6^ average sum-intensity per cell was detected in HeLa cells, which were treated with siRNA against Caveolin-1 (Figure 8). Control cells showed 1.2 × 10^7^ ± 0.1 × 10^7^ sum-intensity per cell and therefore 41% more than cells with a knockdown of Caveolin-1. This effect was statistically significant (γ = 95%, *p* = 0.0019). Depletion of Dynamin 2, Flotillin-1 and Clathrin showed no effect on the detectable fluorescence of SCIONs inside the cells (Figure 8). Although the total amount was slightly increased for all three knockdowns, this effect is statistically irrelevant and can be ignored.

#### Endocytosis mediated by CDC42 and PIP5Kα

Knockdown of CDC42 and PIP5Kα was realized through transfection with specific siRNA compositions via the Dharmacon SMARTpool^®^ technology. As described above, cells were incubated with PEGylated SPIONs for 24 h and iron content per cell was determined. To distinguish between nanoparticles inside the cells and nanoparticles, which are attached to the outer cell membrane, control experiments at 4 °C were performed. 0.8 ± 0.1 pg Fe/cell were detected and subtracted from every measurement. Carboxylated SPIONs were not included because of their very similar behavior compared to the PEGylated SPIONs.

**Figure 9:**
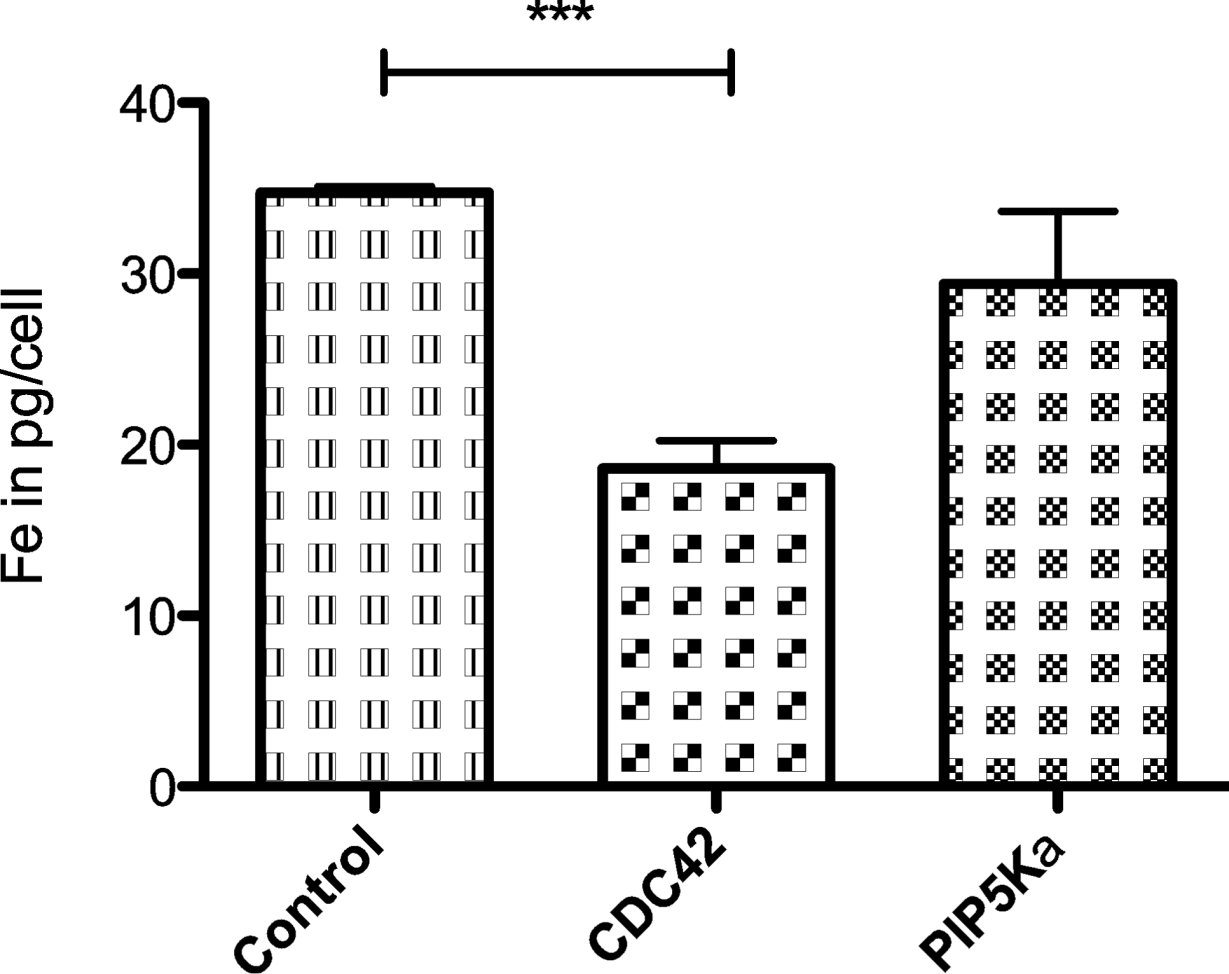
Iron content of control and transfected HeLa cells in pg/cell after 24 h incubation with PEGylated SPIONs; target proteins: CDC42, PIP5Kα (iron concentration 50 µg/mL, error bars: SEM, *n* = 3).

Control cells internalized 34.8 ± 0.4 pg Fe/cell (Figure 9). Depletion of CDC42 decreased this level to 18.6 ± 1.6 pg Fe/cell. Hence, the difference between control cells and cells without CDC42 amounts to 46%. This effect is statistically highly significant (γ = 95%, *p* = 0.0006). Knockdown of PIP5Kα resulted in 29.4 ± 4.2 pg Fe/cell, which is 15% less compared to control cells. But due to the high standard deviation, this effect is not statistically significant (γ = 95%, *p* = 0.2773).

### Summary

Knockdown of Caveolin-1 decreased the ability of HeLa cells to internalize nanoparticles. Depending on the surface modification of SPIONs or SCIONs and the experimental design, the endocytosis of nanoparticles dropped between 23% and 41% compared to non-transfected control cells. Depletion of CDC42 resulted in a reduced endocytosis of PEGylated SPIONs by 46% compared to control cells. Knockdown of other target proteins like Dynamin 2, Clathrin, Flotillin-1 and PIP5Kα did not show significant effects on the internalization behavior of HeLa cells in vitro.

## Discussion

The aim of the study was to elucidate, how human cancer cells internalize iron oxide nanoparticles with silica shells, which have no target function for a special application or receptor. Therefore the human cervical cancer cell line HeLa was chosen as a model cell line. Hela cells are a well-established malignant cell line, which was widely used to study the uptake of iron oxide nanoparticles [18,21,24,32], gold nanoparticles [33–34] and other particle systems like quantum dots [35] or polymer particles [36–37]. To gain insights into the molecular mechanisms, which are involved in the endocytosis of iron oxide nanoparticles, and how the uptake is influenced by parameters like size and surface composition of nanoparticles would be very useful in the development of therapeutic approaches.

### Involvement of Caveolin-1, Flotillin-1 and Clathrin in the endocytosis of SPIONs

The results show, that knockdown of Caveolin-1 reduced endocytosis of unmodified, carboxylated and PEGylated SPIONs by HeLa cells between 23 and 33% compared to control cells (Figure 4, Figure 5 and Figure 6). Therefore this effect was reproducible when SPIONs with comparable properties in size and surface charge were used. The effect may be even more distinct under complete knockdown conditions of Caveolin-1. Although comparison of different particle species is very difficult due to their varying properties, the involvement of Caveolin-1 in the endocytosis of nanoparticles by HeLa cells is consistent with the literature. It was shown, that polyethyleneimine gold nanoparticles around 40 nm [33], gold nanoparticles of 4.5 nm [34] and conjugated polymer nanoparticles [36] are internalized through Caveolin dependent pathways. The same was observed for human alveolar epithelial cells and polystyrene nanoparticles around 100 nm [38] as well as polymer coated gold nanoparticles with a core size around 13 nm [39]. On the other hand there are studies showing the uptake of different nanoparticles by HeLa cells such as quantum dots [35], PEG-PLA particles [37] and mesoporous silica particles [40] exclusively through Clathrin mediated endocytosis and/or macropinocytosis. This discrepancy can be explained by the different physicochemical properties of the particles used. Especially in the field of iron-oxide nanoparticles more studies concerning the endocytotic pathways have to be done to clarify the underlying principles.

Significant influences of Flotillin-1 and Clathrin were not detectable. However, when the cells with knockdown of Flotillin-1 and Clathrin were incubated with SPIONs for 24 h, their intracellular iron content was slightly increased compared to the control cells (Figure 5, Figure 6). This points to a possible compensatory upregulation of other endocytotic mechanisms, as it was shown for HeLa cells [41] as well as for MDCK and HeLa cells incubated with PEG-PLA nanoparticles [37,42].

### Involvement of Caveolin-1, Flotillin-1, Clathrin and Dynamin 2 in the endocytosis of SCIONs

To verify the results gained with SPIONs, SCIONs with comparable properties regarding their chemical composition, size and surface charge were tested with confocal fluorescence microscopy. Dynamin 2 was included as a new target protein because Dynamin 2 has been identified to act together with Caveolin-1 and in other routes of endocytosis [27,43].

The results show, that knockdown of Caveolin-1 decreased endocytosis of SCIONs by HeLa cells by 41% within 4 h incubation time, while knockdown of Flotillin-1 and Clathrin showed no significant effects (Figure 8). This confirms the previous findings regarding the endocytosis of SPIONs. The effect of Caveolin-1 in the endocytosis of SCIONs is higher compared to SPIONs. On the other hand this is possibly due to the different experimental setups, on the other hand SCIONs provided a narrow size distribution, which could support intracellular uptake through distinctive pathways. Potentially the heterogeneous size distribution of SPIONs is also the reason for the relatively low effect of Caveolin-1 compared to other findings in HeLa cells [33–34].

Interestingly, knockdown of Dynamin 2 showed no effect on the endocytosis of SCIONs by HeLa cells. This could be an indication for an unknown, alternative uptake mechanism, which is dependent on Caveolin-1 but independent from Dynamin 2. Because it is known, that Dynamin 2 plays an important role in the constriction of caveolae-coated vesicles from the inner cell membrane [27,43], another possible explanation is, that SCIONs accumulate in caveolae-coated vesicles at the cell membrane without their detachment when Dynamin 2 is depleted. These SCIONs would not have been removed before quantitative analysis. Further experiments have to be conducted to test these hypotheses.

### Involvement of CDC42 and PIP5Kα in the endocytosis of SPIONs

The observed effect of Caveolin-1 on the endocytosis of SPIONS and SCIONs by HeLa cells did not fully explain, how these particles are taken up. So other candidates of the endocytotic machinery had to be tested. Knockdown of CDC42 reduced endocytosis of PEGylated SPIONs by 46% (Figure 9). This effect was highly significant and visible by eye before quantitative iron analysis because of the light-colored cell pellet. The important role of CDC42 is also interesting in the context of the observed effect of Caveolin-1, because it was shown, that depletion of Caveolin-1 in the epithelial ovarian hamster cell line CHO-K1 enhances fluid phase endocytosis dependent on CDC42 [44]. This indicates a possible compensation of the Caveolin-1 knockdown in the experiments with SPIONs and SCIONs. CDC42 is not only related to Flotillin-1, it is involved in many other cellular processes including macropinocytosis [45], therefore explaining its relevance in the uptake of polydisperse SPIONs. Depletion of PIP5Kα caused no significant effect.

## Conclusion

This study shows for the first time, that Caveolin-1 and CDC42 play an important role in the endocytosis of SCIONs and SPIONs with negative surface charge and a primary diameter around 17 to 30 nm in HeLa cells in vitro. Depending on the nanoparticle used, 69 to 87% in addition of the endocytosed particles were taken up through Caveolin-1 and CDC42 dependent pathways. Because of the heterogeneous nanoparticle suspensions, involvement of more than one specific pathway is not surprising. Endocytosis through Caveolin-1 and CDC42 is characterized by vesicles of 30 to 80 nm [27–28], which excludes bigger agglomerates from uptake. For future experiments monodisperse and well-defined particle species would be of special interest for better control of particle properties.

To obtain a deeper understanding of the signaling network underlying the uptake of SCIONs and SPIONs by tumor cells approaches like chemical inhibition of distinct endocytotic pathways, colocalization of particles with key-structures of the endocytotic machinery in fluorescence and electron microscopy or overexpression and dominant negative mutants of key-proteins would be very useful.

General tendencies could be deduced, if these findings are transferable to other human cancer cell lines. But preliminary experiments with the human mammacarcioma cell line BT20 showed no accordance to the findings in HeLa cells (data not shown). More experiments with different cells of different origin have to be conducted to provide more evidence, how cells internalize SPIONs and SCIONs.
